# Mineral Fabric as a Hidden Variable in Fracture Formation in Layered Media

**DOI:** 10.1038/s41598-020-58793-y

**Published:** 2020-02-10

**Authors:** Liyang Jiang, Hongkyu Yoon, Antonio Bobet, Laura J. Pyrak-Nolte

**Affiliations:** 1 Department of Physics and Astronomy, Purdue University, West Lafayette, Indiana 47907 USA; 2 Geomechanics Department, Sandia National Laboratories, Albuquerque, New Mexico 87123 USA; 3 Lyles School of of Civil Engineering, Purdue University, West Lafayette, Indiana 47907 USA; 4 Department of Earth, Atmospheric and Planetary Sciences, Purdue University, West Lafayette, Indiana 47907 USA

**Keywords:** Hydrology, Geophysics, Tectonics, Structural geology

## Abstract

Two longstanding goals in subsurface science are to induce fractures with a desired geometry and to adaptively control the interstitial geometry of existing fractures in response to changing subsurface conditions. Here, we demonstrate that microscopic mineral fabric and structure interact with macroscopic strain fields to generate emergent meso-scale geometries of induced fractures. These geometries define preferential directions of flow. Using additively manufactured rock, we demonstrate that highly conductive flow paths can be formed in tensile fractures by creating corrugated surfaces. Generation, suppression and enhancement of corrugations depend on the relative orientation between mineral fabric and layering. These insights into the role of micro-scale structure on macro-scale flow provide a new method for designing subsurface strategies to maximize potential production or to inhibit flow.

## Introduction

**Figure 1:**
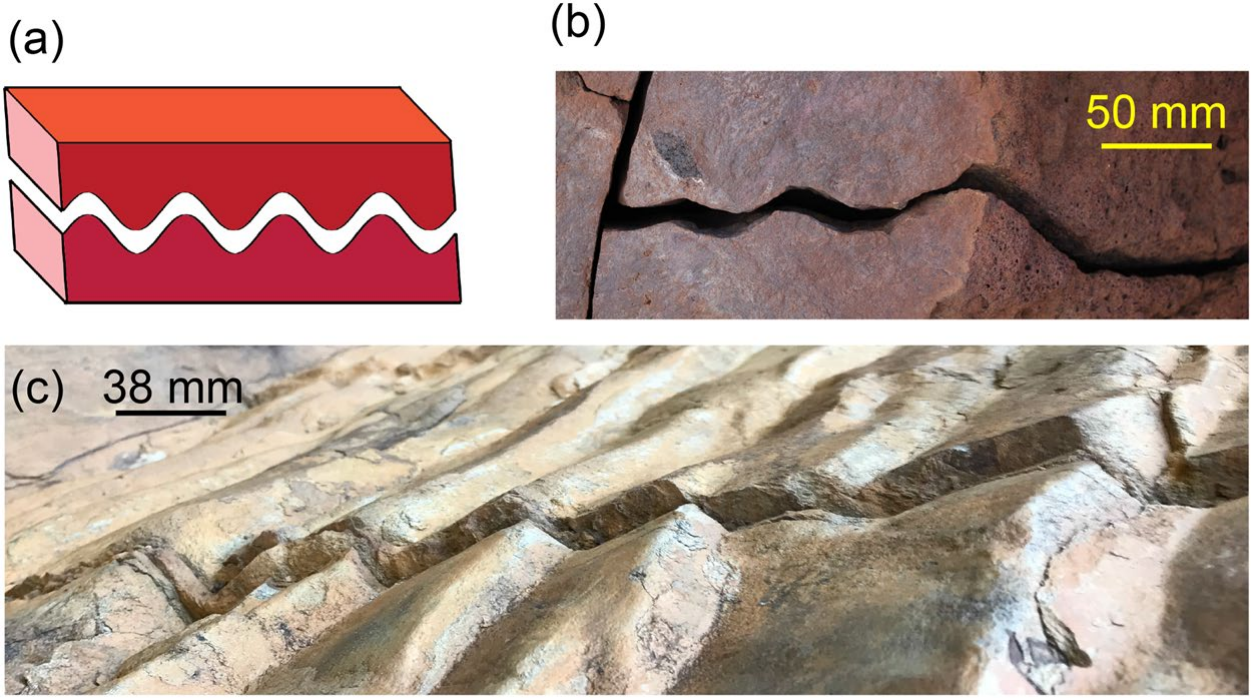
(**a**) Sketch of corrugated fracture surfaces. (**b**) Corrugated fracture in volcanic rock in Hawaii, USA. (**c**) Corrugated fracture surfaces in sedimentary rock in Lederderg Park, Australia. (*Photographs courtesy of Pyrak-Nolte*).

The hydraulic integrity of any subsurface site will be affected by the presence of induced or pre-existing fractures that form highly conductive preferential flow paths. Subsurface flow affects the long-term sequestration of anthropogenic waste, determines the production potential of hydrocarbon reservoir and geothermal energy, and maintains the safety of exploitable aquifers. The conductivity of flow paths is controlled by fracture geometry that can be altered over time from physical and chemical processes^1–4^. When a fracture is generated in rock, two rough surfaces define the void space through which fluids will flow. When corrugated surfaces emerge (e.g. Fig. 1), flow parallel to ridges and valleys is mostly unobstructed compared to the more tortuous path for flow orthogonal to the ridges. Thus knowledge of the presence and orientation of corrugated surfaces enables design strategies for maximizing flow potential.

This raises the fundamental question in fracture mechanics of what gives rise to corrugated surfaces. The roughness of fracture surfaces is known to be affected by mineralogy (mineral fabric, bond strength, spatial distributions), structural features (layers, micro-cracks, etc.), stress orientation, failure mode, and geochemical interactions that can alter mineral bond strength. However, the inherent heterogeneity in mineral phases and composition among rock samples causes a difficulty in identifying the contributions to surface roughness from each of these rock properties and processes, even when extracted from the same rock mass. The spatial variability in compositional and structural features prevents reproducible measurements of fracture formation, deformation, and other physical and chemical properties.

Here, we use additively manufactured gypsum rock to show that mineral fabric orientation governs the isotropy or anisotropy in fracture surface roughness in layered rock which in turn governs the volumetric flow rate through fractures. Through additive manufacturing, the orientation of the mineral fabric and layering can be controlled and used to determine the contributions to fracture surface roughness. The results demonstrate that layer orientation alone is insufficient to predict fracture surface roughness. Knowledge of in-layer mineral fabric orientation is also required. When the resistance to fracturing from layering and mineral fabric orientation acts in the same direction, corrugated surfaces are formed that create highly conductive flow paths parallel to the ridges and valleys of the corrugations. This finding enables the design of fracturing strategies to maximize production potential and provides a method for predicting flow anisotropy in existing fractures through careful examination of in-layer mineral fabric.

## Results

### Geo-architected rock

**Figure 2:**
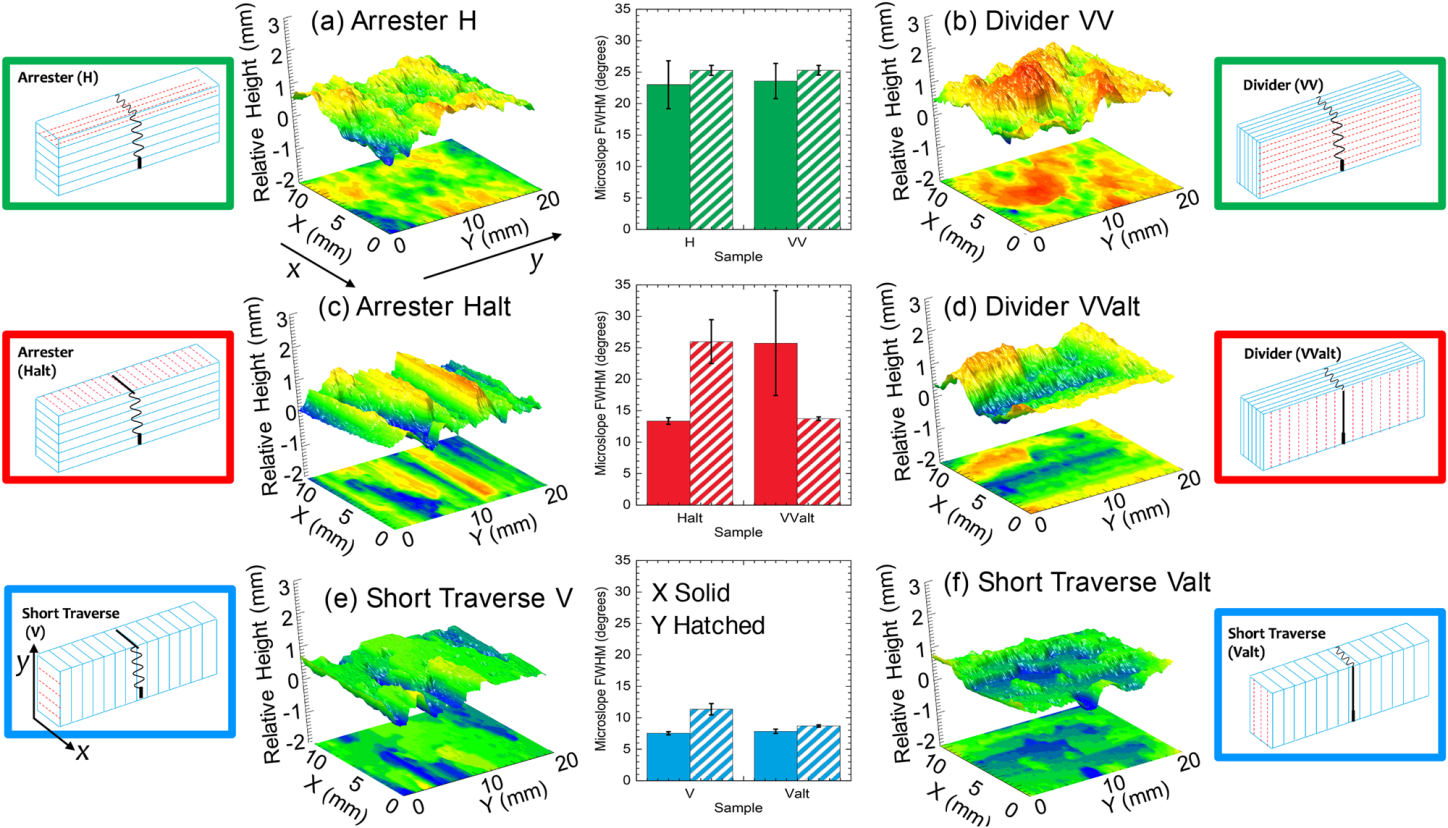
Sketches of sample geometry. Blue lines indicate layers, red dashed lines represent mineral fabric orientation and black lines represent the fracture trace during fracture propagation. Also shown are the a 2D contour plots and 3D surface roughness values for tensile fractures induced in the large samples (**a**) H, (**b**) VV, (**c**) Halt, (**d**) VValt, (**e**) V and (**f**) Valt. In the center, a comparison of the full-width half-maximum (FWHM) of the micro-slope distribution parallel (y-direction hatched shading) and perpendicular (x-direction solid color) to the direction of fracture propagation.

Additive manufacturing can create “geo-architected” rock with repeatable physical and chemical properties to test the hypothesis that in-layer mineral fabric orientation affects fracture surface roughness and macro-scale volumetric flow rates. Samples with different orientations of bassanite (calcium sulfate hemi-hydrate, 2*CaSO*_4_ ⋅ *H*_2_*O*) layers relative to gypsum (*CaSO*_4_ ⋅ 2*H*_2_*O*) mineral fabric were printed (Fig. 2) to examine the effect of fabric direction relative to layer direction on tensile fracture growth and on the geometric properties of the induced fracture surfaces.

The “geo-architected” layered rock samples with preferred mineral fabrics were created using a 3D printing process (see Supplementary Information). Layers of bassanite were bonded with a proprietary water-based binder that produced gypsum as a reaction product. The gypsum mineral fabric direction is oriented by the direction of the binder spreading. When one layer of bassanite is deposited on a previous layer, gypsum crystals form bonds within the layer as well as between bassanite layers after application of the binder. Mineral fabric arises because the gypsum forms stronger bonds between gypsum crystals than between the gypsum crystals and bassanite powder.

**Figure 3:**
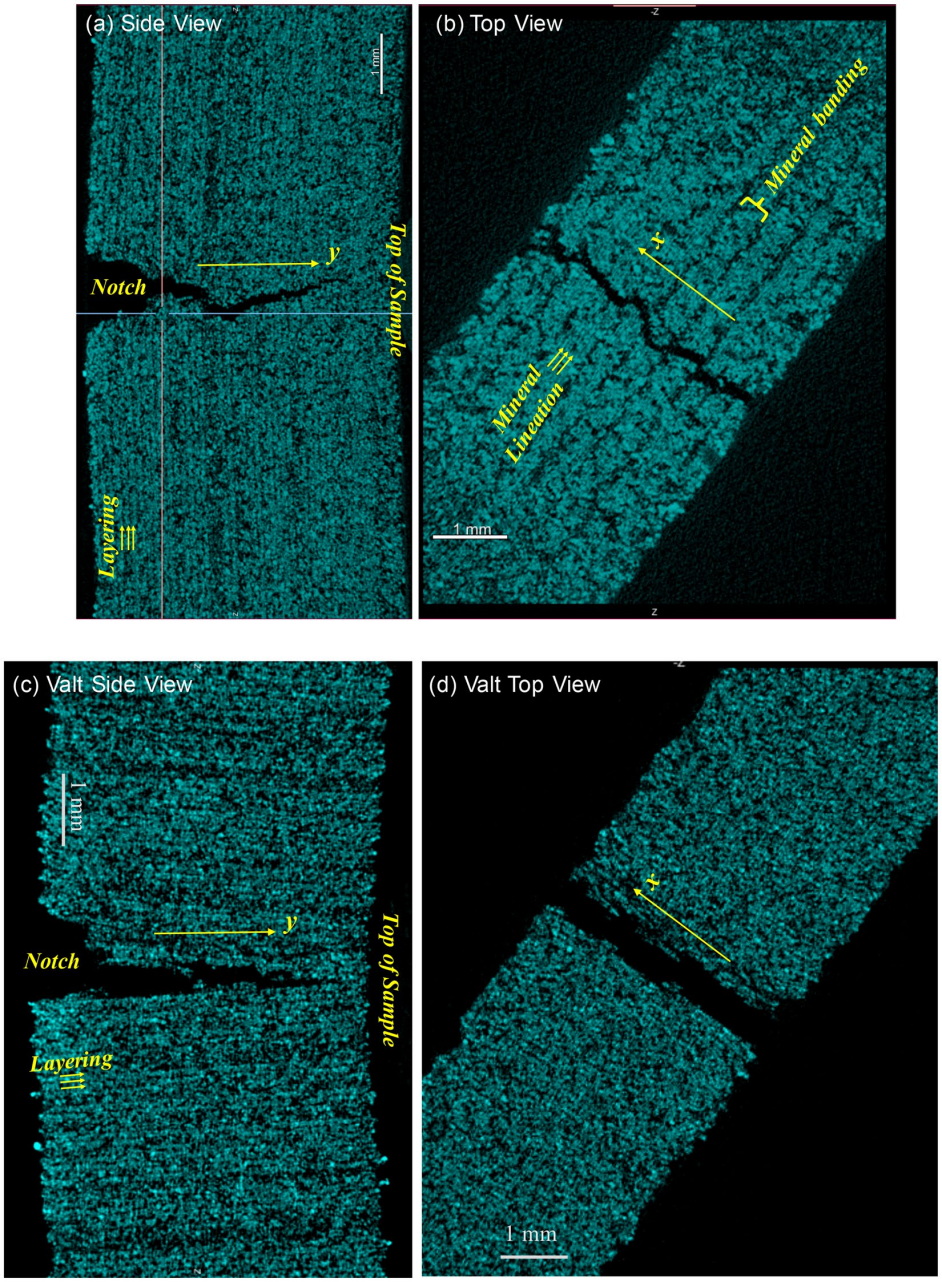
Images from 3D X-ray tomographic reconstruction of small (**a**,**b**) sample H and (**c**,**d**) sample Valt post-peak showing the fracture trace (**a**,**c**) in the side view that shows the fracture trace in the direction of fracture propagation from notch to top of sample (y-direction). In (**a**,**c**), the bassanite layer direction is indicated by the small yellow arrows. The top view for sample H in (**b**) shows the fracture trace in the x-direction and mineral bands and gypsum lineations within the bands. (**d**) The top view of sample Valt contains both layering and the ends of mineral lineations (see Fig. 2f). Scale bars in each image represent 1 mm.

3D X-ray computed microscopy was performed to examine the length scales of the layering and the mineral fabric. Figure 3a,b contains images from a 3D tomographic reconstruction of sample H (Fig. 2a) after post-peak loading, showing the fracture trace in the direction of fracture propagation (y-direction in Fig. 3a) and perpendicular to the direction of propagation (the x-direction in Fig. 3b). In Fig. 3b, mineral bands of gypsum lineations are observed that match the total width (≈500 *μm*) of the spray from the binder application. The gypsum lineations form a preferred mineral fabric orientation. The scale of the gypsum lineations (thickness ≈ 100 *μm*) is on the order of the bassanite layer spacing (≈100 *μm*) (Fig. 3a).

**Figure 4:**
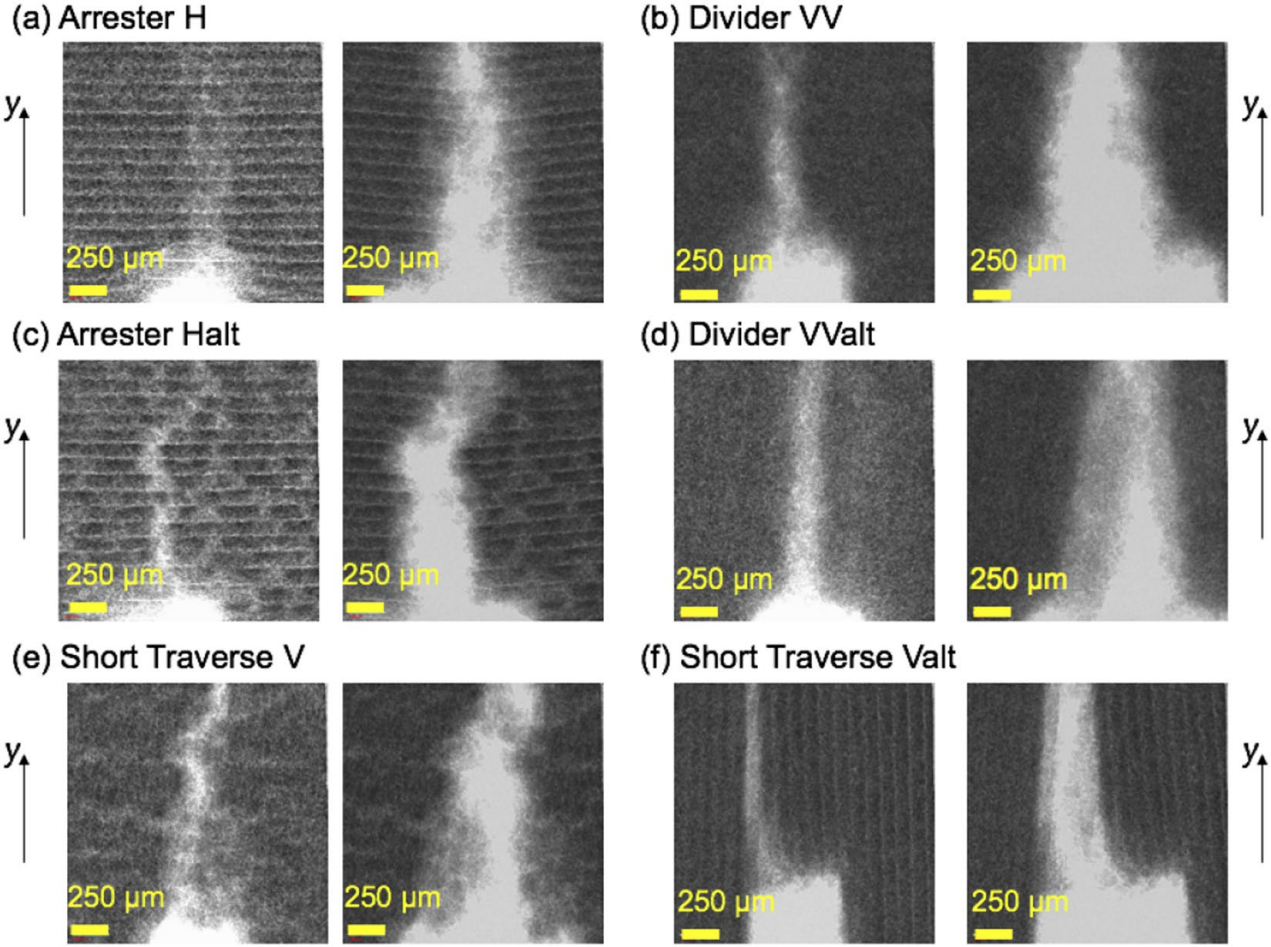
2D X-ray radiographs of the small geo-architected samples at 5% of peak load (left) and just prior to complete failure (right). The direction of fracture propagation from the notch (at the bottom of each image) is in the y-direction. The x-direction is into the page.

The geo-architected rock exhibits anisotropic mechanical properties as determined from ultrasonic measurements of compressional and shear wave velocities (See Supplementary Information). Anisotropy in these samples arises from two sources: (1) the formation of bassanite layers from the successive deposition of bassanite powder during manufacturing; and (2) the direction of mineral fabric which is controlled by the binder application direction. The samples exhibit orthorhombic anisotropy, similar to behavior observed in rock with preferred crystallographic or shape orientation^5^. Single tensile fractures were induced for 4 cohorts of geo-architected rocks (each cohort contained all 6 geometries shown in Fig. 2) using a 3 point bending method (3PB see Methods). The 3PB tests were performed on small samples (4.8 × 25 × 4.2 mm^3^) in-situ in a 3D X-ray microscope to enable high-resolution imaging of the fracture trace during fracture propagation (Figs. 3 and 4), and ex-situ 3PB experiments on larger samples (25.4 × 76.2 × 12.7 mm^3^) to enable quantification of surface roughness and peak failure load. After inducing a fracture in a large sample, laser profilometry was performed to measure the fracture surface roughness of the induced tensile fracture. Spatial correlation lengths, asperity height distributions and micro-slope distributions were calculated from the surface roughness data.

### Fracture topology

Induced fracture propagation in geologic materials depends on the relative resistance to failure among the rock constituents, on the bonding among the constitutents, on mineral cleavage planes, as well as on structural features. Layering and mineral fabric can cause a fracture to wander or deviate from a straight path, creating roughness along the fracture surfaces. This is observed in the 3D X-ray reconstruction for sample H shown in Fig. 3a,b where the fracture is observed to deviate from a straight path because of the layering, and because of the mineral bands and lineations in Fig. 3b. The fracture trace exhibits large-scale roughness on the scale of the width of the mineral banding and finer-scale roughness on the scale of the layers and gypsum lineations, as the competition in resistance to fracturing between the layers and mineral fabric affects the roughness of the induced tensile fractures. In addition, the in-layer orientation of the mineral fabric relative to the layers affects the length scales observed in the surface roughness. In contrast, from the 3D X-ray reconstructions for sample Valt, a relatively straight fracture trace is observed (Fig. 3c,d) with deviations on the scale of the layer thickness (~100 *μ*m) or individual mineral lineations (~100 *μ*m). Mineral banding for sample Valt is parallel to the direction of fracture propagation (Fig. 2f) and does not produce the large scale roughness as observed for Sample H (Fig. 2a).

These trends are also observed from a comparison of fracture traces in X-ray CT images from the different geo-architected samples (Fig. 4). The fracture trace is relatively straight when fractures propagate parallel to the layers (e.g. Valt in Fig. 4f) and deviate from a straight path when propagating across the layers (e.g. H and Halt in Fig. 4a,c). On the other hand, the fracture trace for sample V is not as straight as that observed for sample Valt even though these two samples have the same layer orientation. The difference between V and Valt is the in-layer orientation of the mineral fabric. Laser profilometry was performed on the entire fracture surface from each large sample to provide a more detailed analysis of the fracture geometry and of the impact of mineral fabric orientation on fracture surface roughness.

**Figure 5:**
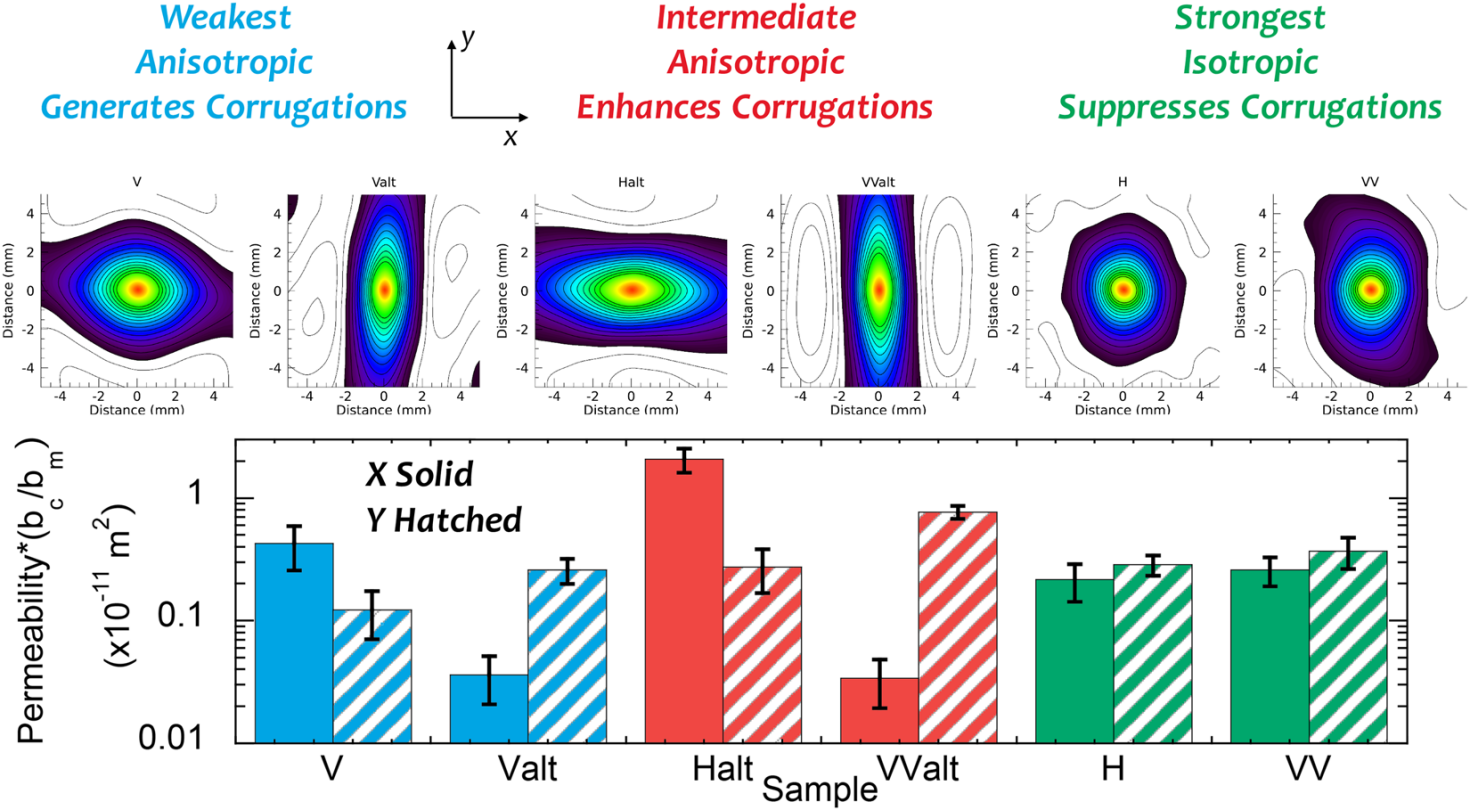
Top row: Normalized 2D autocorrelation function for the geo-architected samples. Bottom row: Averaged numerical fluid permeability based on surface roughness data. Solid color: Flow in the direction perpendicular to fracture propagation (*x-direction*); Hatched/shaded Color: Flow in the direction parallel to fracture propagation (*y-direction*).

The fracture surface roughness from each layer-mineral orientation geometry is shown in Fig. 2 along with the results from a micro-slope, *θ*_*save*_, analysis for the large samples to determine the relative roughness of the surfaces (see Supplementary Information). The results of a 2D autocorrelation analysis are used in Fig. 5 to examine the anisotropy in surface roughness. Whether an induced tensile fracture is corrugated or not (Fig. 2) depends on both the layering and the mineral fabric directions relative to the direction of fracture propagation (y-direction). Fractures are observed to be smooth (*θ*_*save*_ < 15^*o*^ in Fig. 2) but anisotropic when a fracture propagates parallel to the layering (V and Valt in Fig. 5). The anisotropy is caused by the creation of low-amplitude corrugations with the ridges of the corrugations parallel to the mineral fabric orientation (sample geometry inset in Fig. 2e,f). Samples Halt and VValt exhibited strongly anisotropic fracture surfaces with high-amplitude corrugations (i.e. rough surfaces) that ran parallel to both the mineral fabric and the layer orientation (sample inset in Figs. 2c,d and 5). The corrugations were enhanced in these samples because the layering and mineral fabric provided resistance to fracturing in the same direction (Fig. 2c,d). Isotropic rough surfaces with no corrugations were formed in samples H and VV as indicated by the nearly circular contour lines in Fig. 5. For fractures in H and VV type samples, the surfaces were rough (*θ*_*save*_ > 15^*o*^ in Fig. 5) both parallel and perpendicular to the direction of fracture propagation (Fig. 2a,b), leading to isotropic rough surfaces (Fig. 5). When the mineral fabric and layers provide resistance to failure in orthogonal directions, corrugations in fracture surfaces are suppressed (Fig. 2a,b).

### Fluid flow

Fluid flow through a fracture is intimately related to the roughness of the fracture surfaces and the flow path topology that is formed when the two surfaces are placed in contact^2,6^. Simulations of fluid flow were performed using the surface roughness measurements from each sample and the numerical method described in^2,6–8^. The permeability is scaled by the ratio of the critical neck (smallest aperture along the dominant flow path) to the mean aperture (for values see Supplementary Information). With this normalization, variations in flow rate are related to spatial correlations in the aperture distribution^2^.

Samples with isotropic surface roughness (H & VV) exhibited average flow rates in the two orthogonal directions that were within 30%. For anisotropic surfaces (V, Valt, Halt and VValt), average flow rates varied between the parallel and perpendicular directions of fracture propagation by factors of 4 to 40. For Halt and Valt (the samples exhibiting the strongest corrugations in surface roughness) the permeability was greater parallel to the ridges than perpendicular to the ridges. Therefore, the anisotropy observed from the microslope and autocorelation analyses (Fig. 2) is associated with the permeability anisotropy for the fractures (Fig. 5). This finding suggests that estimates of macroscale permeability isotropy or anisotropy could potentially be predicted from microscale study of mineral fabric orientation relative to layering and relative compositional bonding strengths prior to fracturing a rock

## Discussion

The hydraulic, mechanical and seismic responses of a fracture are controlled by the geometry formed from two rough surfaces in contact. This geometry forms the basis of a scaling relationship between fluid flow through a fracture and fracture specific stiffness^2^. Fracture specific stiffness is an effective parameter that captures the deformed topology of a fracture^8^. Given the centrality of fracture geometry to this scaling relationship, a key question is what controls this fracture geometry.

Previous studies on rock and analog rock have shown that crack initiation and propagation are affected by tensile versus shear failure that affect fracture geometry (e.g.^9^). Past and current research has also shown that fracture toughness, *κ*, i.e., the ability of a material to resist fracturing, is affected by layer orientation, with the geometry of the layers referred to as arrester (e.g. H & Halt), divider (e.g. VV & VValt) and short traverse (e.g. V & Valt)^10^ (Fig. 2). For shale, many studies have observed that fracture toughness is ranked by the orientation of the layers with the toughness sequence: *κ*_*divider*_ > *κ*_*arrester*_ > *κ*_*shorttraverse*_^11,12^. However, other studies on shale have observed cases where fracture toughness is comparable between *κ*_*arrester*_ ≈ *κ*_*divider*_ or between *κ*_*arrester*_ ≈ *κ*_*shorttraverse*_, or even that values of fracture toughness for the short traverse specimens exhibit both the highest and lowest values^13^. These differences from the expected ranking of fracture toughness have been attributed to percent kerogen, inelasticity, clay, variable elastic properties among layers in shale, and also microfractures^10,13–15^. These studies demonstrate the difficulty working with natural geologic materials and suggest that micro-scale compositional and structural properties play a strong role in macro-scale measurements of resistance to fracturing.

**Figure 6:**
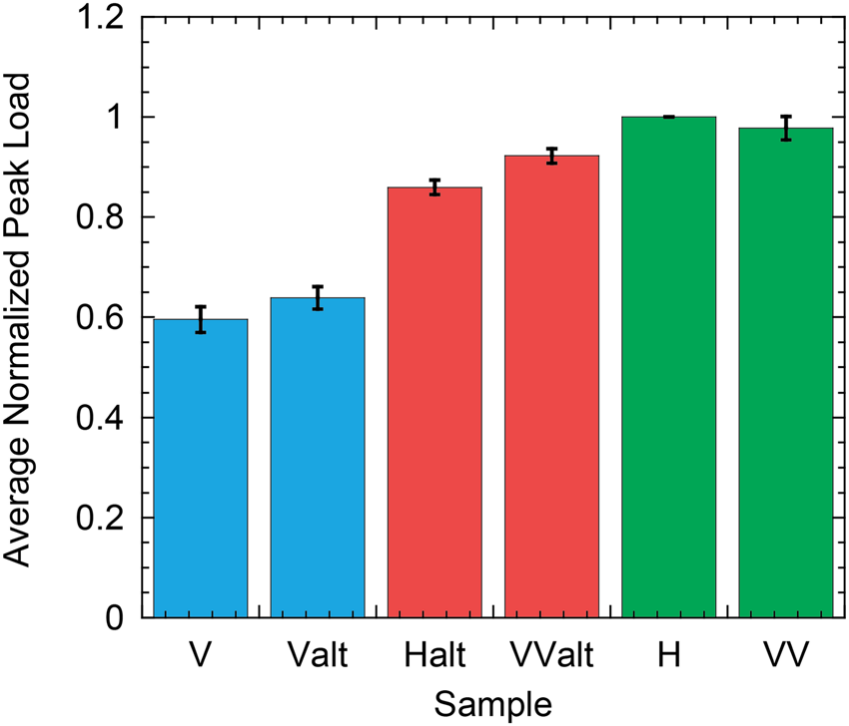
Average relative peak load from 4 cohorts of samples for the geo-architected samples (values are relative to H samples). The samples are color-coded to match the colors in graphs of permeability and microslope analysis (Figs. 2 and 5, respectively).

The recent advancements in the creation of “geo-architected” rock through additive manufacturing provide a method for creating highly reproducible samples with specific aspects of geologic material to enable identification of physical properties that contribute to the complexity of fracture formation in natural media. Here, the role of layering and in-layer mineral fabric orientation on the formation of fractures was examined using “geo-architected” rock with designed in-layer mineral orientation and designed layer geometries. Previous studies on shale focused on the resistance to failure as a function of layer orientation but observed no clear trends. From our study, the co-existence of layers with in-layer oriented mineral fabric enabled identification of one feature of geologic media that can explain the inconsistency observed in natural rock. From mechanical testing on the geo-architected samples, the peak failure load differed among the geo-architected samples, even for samples with the same layer orientation but different mineral fabric orientation. For example, the peak failure load, *F*_*peak*_, for arrester samples, H and Halt, differed though both contained layers that were oriented perpendicular to the loading (Fig. 6). *F*_*peakH*_ > *F*_*peakHalt*_ because the fracture in H broke across the gypsum crystals (Fig. 2). The bonds between gypsum crystals (located between sequential bassanite layers) were stronger than the bonds between the gypsum and bassanite. The weakest geo-architected samples were the short traverse samples, V and Valt (Fig. 6), both of which contained layers parallel to the direction of fracture propagation. Thus the resistance to fracturing arises from layer orientation as well as in-layer mineral fabric orientation.

As observed in Figs. 3 and 4, the fracture trace geometry is affected by layering and in-layer mineral orientation as both contribute to the resistance to cracking and can cause the propagating fracture to deviate from a straight path. A link is observed between the resistance to failure and the roughness of the induced tensile fractures for the geo-architected samples (Figs. 5 and 6). If a propagating fracture crosses the layering, the fracture surfaces tend to be rough in one (Fig. 2c,d) or two (Fig. 2a,b) directions. Isotropy is observed in samples H and VV because the resistance to failure from the layering and the in-layer mineral fabric act in orthogonal directions and produce roughness on the same scale; in this case, the scale of the thickness of the bassanite layer and the scale of the gypsum lineations (Fig. 3). For these isotropic surfaces, corrugations are suppressed, resulting in an isotropic fracture permeability (Fig. 5).

Corrugated surfaces were produced under two conditions: (1) when a fracture propagated parallel to layering but deviated around oriented mineral fabric (V and Valt in Fig. 2e,f); and (2) when the resistances to cracking from the mineral fabric and layer orientations were aligned (Halt and VValt in Fig. 2c,d). For both conditions, the ridges of the corrugations always ran parallel to the mineral lineations (red lines in Fig. 2). The first condition was observed in samples V and Valt where the resistance to failure was low (Fig. 6). The fracture surfaces tended to be smooth and the anisotropy was governed by the mineral fabric orientation that generated low-amplitude corrugations. The low-amplitude corrugations were sufficient to produce anisotropic flow (Fig. 5) with the dominant flow direction parallel to the ridges of the corrugations. The second condition was observed for samples Halt and VValt where high-amplitude corrugations were observed on the scale of the mineral bands (Fig. 3b) and the ridges of the corrugation were also aligned parallel to the direction of mineral fabric (red lines in Fig. 2c,d). The corrugations led to strong anisotropy in fracture permeability (Fig. 5). The strong anisotropy in the asperity height distribution for Halt and VValt occurred because the layers and mineral fabric each provided geometric toughening in the same direction, enhancing the roughness. These results indicate that an additional toughness is associated with the difference in resistance to fracturing of the layering relative to the minerals, and this difference affects fracture surface roughness.

The ability to predict the existence and orientation of corrugations in fracture surface roughness is of key importance for subsurface activities that inject or withdraw fluids from the subsurface. As observed from the fluid flow analysis, flow parallel to corrugations is larger than across corrugations (Fig. 5). The largest flow rates were found for samples where the in-layer mineral fabric orientation enhanced the corrugations (samples Halt & VValt) because the resistance to fracturing from the mineral fabric and layering were aligned producing corrugations as shown in the sample sketches in Fig. 2c,d and observed in the surface roughness.

In this study, only tensile failure of fractures was considered but conditions in the subsurface may vary because of local stresses, well orientation and fluid pressure. The same crack in the field, with far-field geostatic stresses would be subjected to mixed-mode loading conditions if the crack is not aligned with the principal directions of the geostatic stresses. However, the resistance to failure is expected to still play a dominant role under these conditions. While the results presented in the paper are obtained from Mode I (opening) loading, recent tests on mixed mode loading (Modes I and II, opening and shearing) seem to indicate that the roughness of the induced fracture is still controlled by the fabric. These findings suggest that there is an opportunity to drill horizontal wells in a direction that tunes the roughness of the induced cracks to maximize flow, given the orientation of the far-field stresses and the rock fabric. However, this may be at the expense of larger breakout pressures. Laboratory-scale hydraulic fracturing tests have demonstrated that rock fabric plays a role in the induced fracture complexity and containment height^16^. For fluid-driven cracks, such as in hydraulic fracturing, the same mode I loading would occur (neglecting possible corrosion/chemical, leakoff and injection rate effects) if the crack is aligned with the principal directions of the geostatic stress. Otherwise, the fluid-driven crack would also be subjected to mixed-mode loading conditions as described above with the same results and potential to design fracture roughness to enhance flow.

The ability to predict corrugations from layer orientation alone is only possible when there is no preferred mineral orientation or no difference in bond strength between the constituent minerals. In this study, the difference in strength among the bassanite-gypsum and the gypsum-gypsum bonds affected the resistance to fracturing which in turn affected the fracture surface roughness. In natural geologic media, it is well known that minerals can contain preferred planes of weakness (cleavage planes) from differences in bonding in one or more directions, and from preferred mineral orientations (foliations) that are caused by physical forces during formation such as sedimentation, magma flow or shearing. The potential for oriented minerals within a layer has been observed in igneous rocks such as gabbro that exhibit obliquity between foliation and compositional layering^17^, schist and gneiss, where the foliation and fold axial planes are not aligned^9^ and in clay in shales^5^. The results presented here suggest that detailed mineralogical studies of cores should be performed to aid interpretation of preferred flow paths in existing fractures and to aid the design of induced fractures to maximize production potential.

## Methods

### Samples

For the ex-situ three-point bending (3PB) tests, the dimensions of the large printed specimens were 25.4 × 76.2 × 12.7 mm^3^ with a 5.08 mm long by 1.27 mm wide central notch to induce tensile failure. The notch was formed during printing, For experiments performed with the in-situ stress rig in the 3D X-ray microscope, small 3PB samples were printed with a height of 4.8 mm, a length of 25 mm and a width of 4.2 mm and contained a printed central notch with a height of 0.96 mm.

### Inducing fractures

Tensile fractures (Mode I) were induced in samples using the three point bending test (e.g.Yu *et al*.^18^). A rod was placed on the top surface of a sample directly aligned with the notch (Fig. 2) and two rods were placed symmetrically on the bottom surface at a distance of 10% of the sample length (7.6 mm for the large samples and 2.5 mm for the small samples, *z-direction in a right-handed system* in sample sketch in Fig. 2e) from the sides of the sample. Load was applied to a sample using an ELE International Soil Testing load frame with a 8900 N capacity S-shaped load cell. The loading rate was 0.03 mm/min. Load and displacement (from a LVDT) data were recorded at a 5 Hz recording rate.

### Fracture roughness

Surface roughness measurements were made with a Keyence LK-G152 Laser with a wavelength of 650 nm and a laser spot size was 120 *μm*. The sample was mounted on coupled orthogonal translation stages (Newport MTM250PP1) controlled by a motion controller (Newport Universal Motion Controller ESP 300) to measure asperity height over a 2D area (10.5 mm by 20.0 mm) in increments of 0.1 mm which enabled slight oversampling to enhance the signal to noise.

### Flow simulation

The fracture geometry for the flow simulations was based on the asperity height distribution determined from laser profilometry measurements. The flow was calculated for each subregion of each fracture in each cohort. The same subregions were used in the autocorrelation analysis. By placing a rough surface against a flat plane^19,20^ that creates 5% contact area, a uniform reference point was created to compare the effect of large-scale roughness (i.e. corrugations) among the different fracture surfaces. The aperture was assumed to be proportional to the surface roughness. Using the approach of^8^ and^2^, a network of elliptical pipes from the inlet to the outlet was created. The resistance of each elliptical pipe depended on the major and minor axes of the two ellipses that defined each pipe. Additional details on the approach can be found in^2,6,7^ and in the Supplementary Information. The calculated permeability was normalized by the ratio of the aperture of the critical neck, *b*_*c*_, to the mean aperture, *b*_*m*_ to compare the different samples. The values of *b*_*c*_ and *b*_*m*_ are given in the supplementary material.

## Supplementary information

Supplementary Information.
