# Supplementary material for: Mineral Fabric as a Hidden Variable in Fracture Formation in Layered Media
Source: Sci Rep. 2020 Feb 10;10:2260. doi: 10.1038/s41598-020-58793-y (PMC7010730; doi:10.1038/s41598-020-58793-y)
Supplement: Supplementary file 1 — Supplementary Information. [file 41598_2020_58793_MOESM1_ESM.pdf]

Supplementary Information: Mineral Fabric as a Hidden Variable in Fracture Formation in Layered Media

L. Jiang[1], H. Yoon[2], A. Bobet[3], and L.J. Pyrak-Nolte*[1,3, 4]

[1] *Department of Physics and Astronomy, Purdue University, West Lafayette, Indiana*
[2] *Geomechanics Department, Sandia National Laboratories, Albuquerque, New Mexico*
[3] *Lyles School of Civil Engineering, Purdue University, West Lafayette, Indiana*
[4] *Department of Earth, Atmospheric and Planetary Sciences, Purdue University, West Lafayette, Indiana*

January 28, 2020

*Corresponding Author: ljpn@purdue.edu

1 Supplementary Information

1.1 3D Printing

Geo-architected layered rock samples with preferred mineral fabrics were created using a ProJet CJP 360 3D printer. Layers of calcium sulfate hemi-hydrate (0.1 mm thick bassanite powders, Figure 1 left) were bonded with a proprietary water-based binder (ProJet X60 VisiJet PXL) that produced gypsum as a reaction product (Figure 1 center). The gypsum mineral growth direction is oriented by the direction of linear movement by the binder spray head that moves in a manner similar to an ink jet printer. The width of the mineral bands (see Figure 3b in the paper) is controlled by the design of the binder spray head which contains evenly spaced holes to release the binder. After one layer of bassanite is deposited, the binder is sprayed across the surface in linear rows. When a new layer of bassanite is deposited on the previous layer, the reaction of binder with bassanite powders causes gypsum crystals to form bonds between the bassanite layers. The direction of the binder spray head is an input parameter to the 3D printer. The sample geometry is designed in stereolithography CAD software (i.e. STL format) that is imported into 3D printing software. During the layout in the CAD software, the samples are oriented to produce the mineral fabric orientation shown in Figure 2 in the manuscript.

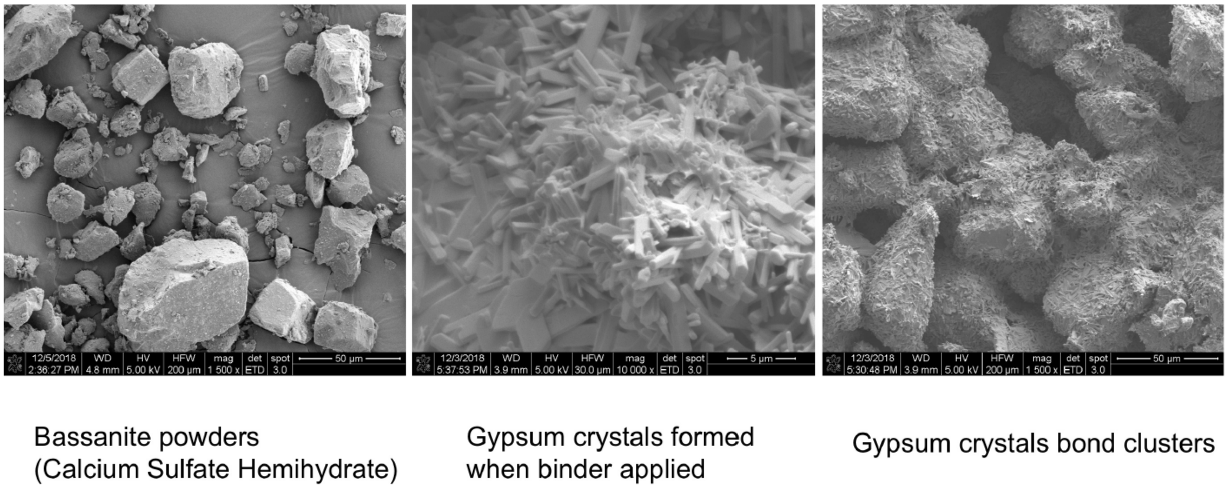

Figure 1: Scanning Electron Microscopy (SEM) images of (left) bassanite powder, (center) gypsum crystals formed from the binder application, and (right) clusters of gypsum crystals.

1.2 Material Properties

Powder X-ray diffraction (XRD) was performed to determine the percent bassanite ($2CaSO_4 \cdot H_2O$) and gypsum ($CaSO_4 \cdot 2H_2O$) in the 3D printed samples. The XRD system was a Panalytical Empyrean X-ray diffractometer equipped with Bragg-Brentano HD optics, a sealed tube copper X-ray source ($\lambda = 1.54178\mathring{A}$), with soller slits on both the incident and receiving optics sides, and a PixCel3D Medipix detector. The anti-scatter slit ($1/2^o$) and divergence slit ($1/8^o$) as well as the mask (4 mm) were chosen based on sample area and starting θ angle. The XRD measurement found that the powder contained 97% bassanite while the printed samples (i.e. after application of the binder) were ≈50-50 bassanite and gypsum. The average density of the 3D printed geo-architected samples was $1190 \pm 5.5 kg/m^3$.

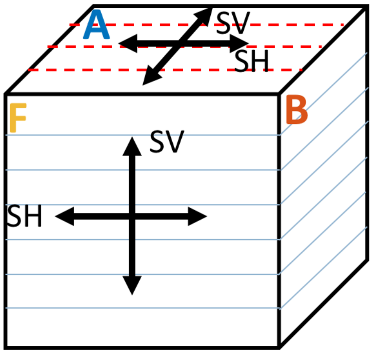

Figure 2: Solid blue lines represent layering. Red dashed lines represent direction of mineral fabric. (Note: *Not drawn to scale.*) Shear wave polarizations are indicated by the black arrows. Waves were propagated from A-to-C, B-to-D and E-to-F.

Compressional and shear wave velocities were determined from ultrasonic waves measurements on a cube of 3D printed geo-architected gypsum samples. Olympus V103 (P-wave) and V153 (S-wave) piezoelectric transducers with a central frequency of 1 MHz were coupled to the sample with baked honey (8.75% weight loss from the removal of water). An Olympus 5077PR pulse generator excited the source with 400V with 0.4 μs width with a 100Hz repetition rate. After propagating through the sample, the signals were digitized using a National Instruments USB-5133 digitizer and stored on a computer for analysis. A sampling rate of 100 MSamples/sec was used to get a bin size of 0.01 microseconds. The dimensions of the cubes and measured velocities are listed in Table 1. For comparison, Table 2 provides compressional and shear wave velocities for a layered shale [1].

Unconfined compressive strength (UCS) testing was performed on 3D printed gypsum samples

Table 1: Compressional (P) and Shear wave (S) Velocity measured on a cubic sample of 3D Printed Rock. Shear wave polarizations, S_H and S_V, are shown in Figure 2.

Waves	Length (mm)	P (m/s)	S_H (m/s)	S_V (m/s)
A-to-C	49.7	2360	1475	1419
B-to-D	49.7	2737	1549	1455
E-to-F	49.3	2430	1539	1381

Table 2: Unconfined compressive strength (UCS), Compressional (P) and Shear wave (S_H) Velocities for shale with bedding[1].

Orientations	UCS (MPa)	P (m/s)	S_H (m/s)
Parallel to bedding	67	3810	2350
Perpendicular to bedding	64.2	3807	3090
Parallel to bedding	70.2	3438	2610

with a diameter of 25.4 mm and a height of 50.8 mm using an ELE International Soil Testing uniaxial loading machine (capacity 8898 N) with a displacement rate of 0.03 mm/min. Load and displacement data were recorded at a 5 Hz sampling rate. Displacement and load were read to within 0.01 mm and 5 N, respectively. Figure 3 shows the geometry of the cylinders used in the UCS testing and representative stress-strain curves for the 3D printed samples. UCS values for the 3D printed geo-architected samples are given in Table 3 and for comparison values for shale are given in Table 2. An additional comparison between 3D printed gypsum samples and natural rock can be found in Kong et al. [2]

Table 3: Average Unconfined Compressive Strength (UCS) for the samples shown in Figure 3.

Sample	UCS (MPa)
H	11.2
T	11.5
V	14.7

1.3 Xray CT

A 3D X-Ray Microscope (Zeiss Xradia 510 Versa) was used to acquire 2D radiographs and to perform 3D computed tomography for the small geo-architected samples during in-situ 3PB loading test. The small samples were placed in a Deben CT5000 in-situ uniaxial loading device in the 3D X-ray microscope. The loading rate was 0.1 mm/min with load information recorded at 100 milisecond sampling rate. The settings for both the 2D and 3D scans were 80 kV and 7W Xrays,

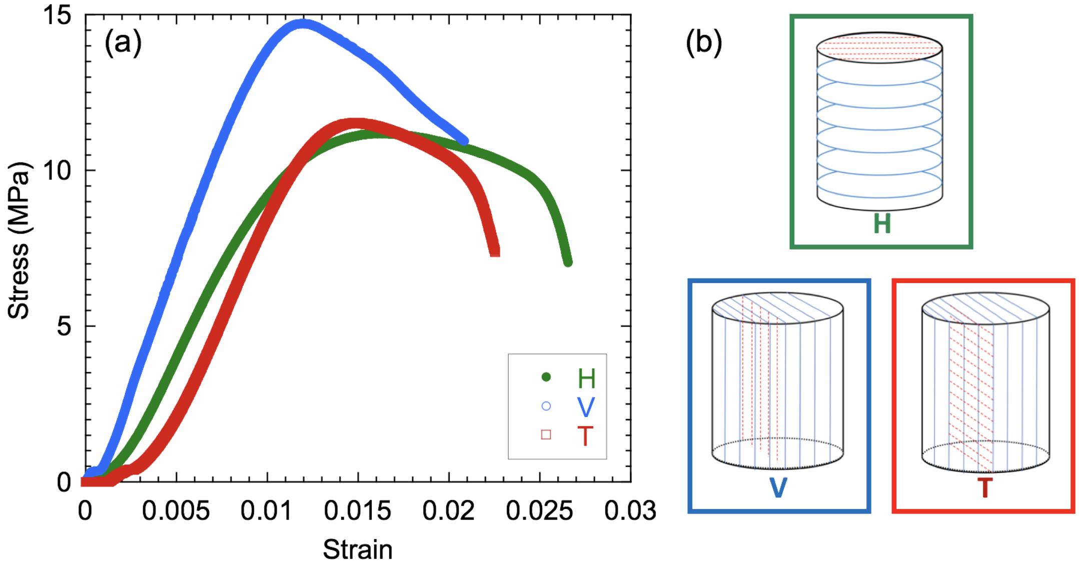

Figure 3: (a) Stress-strain curves for (b) 3D printed geo-architected samples H, V and T with layering (blue lines) and mineral texture (red lines) shown in the sketch of the samples. (Note: Layering and mineral texture are *not drawn to scale.)*

at 4x magnification, with a source and detector distances of 70 mm and 200 mm, respectively, and 4s exposure time for each of the 1601 projections. The voxel and pixel edge length (both for the 3D and 2D scans) was 1.75 μm. The field of view was 1.78 mm^2. Data reconstructions and anlaysis were performed using Object Research Systems (ORS) Dragonfly Pro 4.0 software.

1.4 Surface Roughness Analysis

The surface roughness maps were corrected for arbitrary rotations associated with mounting the sample in the laser profilometery system. The gradients were determined by fitting a 2D plane to the surface and then subtracting the gradients from the asperity arrays. Next, the minimum asperity height was subtracted from all points to yield asperity heights, $z_(x, y)$ that ranged from zero to the maximum for a given surface.

The isotropy or anisotropy of a surface asperity height distribution was determined from a 2D auto-correlation analysis. In this approach, a 2D Fourier Transform, FT, of the asperity heights from a surface, $Z = FT(z(x, y))$, was multiplied by the complex conjugate, $\tilde{Z}$, and then an inverse Fourier transform, FT^{-1}, was performed on this product:

$$S(x,y) = \frac{FT^{-1}(Z * \tilde{Z})}{< z(x,y) * z(x,y) >} \quad (1)$$

and divided by the mean of the square of $z(x,y)$ to obtain $S(x,y)$, the 2D auto-correlation function. The 2D asperity map was rectangular in shape which could bias or generate artifacts in $S(x,y)$. For each surface roughness map, the auto-correlation function, $S(x,y)$, was calculated for 2 circular subregions (10 mm diameter). For each sample type (i.e. 3D printed samples), the presented 2D auto-correlation functions represent an average $< S(x,y) >$ overall samples of a given type. The auto-correlation function indicates the probability that an asperity at a distance $r(x,y)$ will have a similar height. The maximum probability is 1 when $r(x,y) = 0$ when a comparison is made between a point and itself.

Micro-slope angle analysis was also performed on the asperity height map, $z(x,y)$, to serve as a measure of the relative smoothness or roughness. Park & Song [3] defined the microslope angle as the dip of the slope between neighboring asperities. A microslope analysis was performed by finding the local slope, s, where

$$s_x = \frac{dz(x,y)}{dx} \quad (2)$$

and

$$s_y = \frac{dz(x,y)}{dy} \quad (3)$$

which is the derivative of the surface roughness profile in the x-direction (horizontal and perpendicular to the direction of fracture propagation) and y-direction (vertical direction and parallel to the direction of fracture propagation). The microslope angle is taken relative to the horizontal and is found by

$$\theta_{sx} = arctan(s_x) \quad (4)$$

and

$$\theta_{sy} = arctan(s_y). \quad (5)$$

A surface was defined as relatively "smooth" if the average microslope angle distribution full-

width at half the maximum,(θ_{save}), was $\theta_{save} < 15^o$. $\theta_{save} \approx 60^o$ has been measured on induced fractures in granite [4], while for synthetic gypsum fractures cast against sand paper, $\theta_{save} \approx 10^o, 30^o$, and 40° were observed as the grit size increased from 68 to 265 to 530 micrometers [5]. The original work by Park & Song [3] found $\theta_{save} \approx 20^o - 30^o$ for joints in granite and gneiss. From fracture surfaces in slate after shear box testing [6], gaussian distributions of microslope angles were observed that were centered on 5° and -8° for different applied normal loads with $\theta_{save} \approx 15^o$, and 25°, respectively.

1.5 Flow Simulation

The aperture distribution was obtained by placing the measured rough surface against a flat plane that creates 5% contact area to capture the effect of large scale roughness on fluid flow. This yields a NxN array of apertures that is replaced with a network or graph of elliptical pipes[7, 8, 9, 10, 11, 12, 13] from which a sparse system of linear equations of $O(N^2)$ is generated.

For each pipe, the volumetric flow rate is given by $q = \Delta P/R$ where R is the resistance and ΔP is the head. The resistance is given by

$$R = \frac{4f\mu\Delta l\sqrt{K}(K+1)}{a\pi} \tag{6}$$

with f given by

$$f = \frac{a_1 b_1 + a_2 b_2}{2A_{avg}} \tag{7}$$

and $K = \frac{a^2}{h^2}$. The major and minor axes of the two ellipses (which represent the ends of an elliptical pipe) that is formed between adjacent rows of apertures in the array are given by a_i and b_i where $i = 1$ and 2. Half of the maximum aperture is represented by H, while a is the average minor axis between rows. The average area of the two ellipses is given by A_{avg}. Additional details on the approach can be found in Pyrak-Nolte & Morris [14], Cheng et al. [10], and Petrovitch [11].

1.6 Permeability Normalization

The average mean aperture and average aperture of the critical neck in the $x-$ and $y-$ directions are listed in Table 4 and were used to normalize the average permeability for each sample.

Table 4: Average mean aperture, b_m, and average critical aperture, b_c for the $x-$ and $y-$ directions used to normalize the permeability

Sample	Mean Aperture (μm)	Critical Neck x-direction (μm)	Critical Neck y-direction (μm)
H	305	411	538
Halt	490	835	437
V	330	447	491
Valt	359	249	587
VV	262	478	330
VValt	204	241	390

References

[1] Yoon, H. *et al.* Impact of depositional and diagenetic heterogeneity on multiscale mechanical behavior of mancos shale, new mexico and utah, usa. In Camp, W. *et al.* (eds.) *AAPG Memoir Mudstone Diagenesis: Research Perspectives for Shale Hydrocarbon Reservoirs, Seals, and Source Rocks*, 121–148 (AAPG, 2019).

[2] Kong, L., Ostadhassan, M., Li, M. & Tamimi, N. Can 3-d printed gypsum samples replicate natural rocks? an experimental study. *Rock Mech. Rock Eng.* (2018).

[3] Park, J.-W. & Song, J.-J. Numerical method for the determination of contact areas of a rock joint under normal and shear loads. *International Journal of Rock Mechanics and Mining Sciences* **58**, 8–22 (2013).

[4] Choi, M.-K. *Characterization of Fractrue Stiffness subjected to Normal and Shear Stress.* Ph.D. thesis, Purdue University (2014).

[5] Choi, M.-K., Bobet, A. & Pyrak-Nolte, L. J. The effect of surface roughness and mixed-mode loading on the stiffness ratio kx/kz for fractures. *Geophysics* **79**, 1–13 (2014).

[6] Nguyen, V., Konietzky, H. & Fruhwirt, T. New methodology to characterize shear behavior of joints by combination of direct shear box testing and numerical simulations. *Geotech. Geol. Eng.* **32**, 829–846 (2014).

[7] Yang, C., Cook, N. G. W. & Myer, L. R. Network modelling of flow in natueral fractures. In *Rock Mechanics as a Guide for Efficient Utilization of Natural Resources*, 57–64 (1995).

[8] Tran, J. *Efficient Simulations of Multiphase Flow in Three-dimensional Fracture Networks*. M.s. thesis (1998).

[9] Pyrak-Nolte, L. J. & Morris, J. P. Single fractures under normal stress: The relation between fracture specific stiffness and fluid flow. *International Journal of Rock Mechanics and Mining Sciences* **37**, 245–262 (2000).

[10] Cheng, J. T. *et al.* Single-phase flow in a rock fracture: micro-model experiments and network flow simulation. *International Journal of Rock Mechanics and Mining Sciences* **41**, 687–693 (2004). URL `<GotoISI>://000221926200010`.

[11] Petrovitch, C. *Universal Scaling of Flow-Stiffness Relationship in Weakly Correalted Fractures*. Ph.d. thesis (2013).

[12] Petrovitch, C., Pyrak-Nolte, L. & Nolte, D. D. Combined scaling of fluid flow and seismic stiffness in single fractures. *Rock Mechanics And Rock Engineering* (2014).

[13] Pyrak-Nolte, L. & Nolte, D. D. Approaching a universal scaling relationship between fracture stiffness and fluid flow. *Nature Communications* **7**, Article Number 10663 (2016).

[14] Pyrak-Nolte, L. J. & Morris, J. P. Single fractures under normal stress: The relation between fracture specific stiffness and fluid flow. *International Journal of Rock Mechanics Mining Science & Geomechanics Abstracts* **37**, 245–262 (2000).
